# Association of early childhood constipation with the risk of autism spectrum disorder in Taiwan: Real-world evidence from a nationwide population-based cohort study

**DOI:** 10.3389/fpsyt.2023.1116239

**Published:** 2023-03-30

**Authors:** Yi-Feng Lee, Meng-Che Wu, Kevin Sheng-Kai Ma, Jing-Yang Huang, James Cheng-Chung Wei

**Affiliations:** ^1^Division of Neonatology, Children’s Medical Center, Taichung Veterans General Hospital, Taichung, Taiwan; ^2^School of Medicine, Chung Shan Medical University, Taichung, Taiwan; ^3^Division of Gastroenterology, Children’s Medical Center, Taichung Veterans General Hospital, Taichung, Taiwan; ^4^Department of Post-Baccalaureate Medicine, College of Medicine, National Chung Hsing University, Taichung, Taiwan; ^5^Department of Epidemiology, Harvard T.H. Chan School of Public Health, Boston, MA, United States; ^6^Center for Global Health, Perelman School of Medicine, University of Pennsylvania, Philadelphia, PA, United States; ^7^Department of Orthodontics and Dentofacial Orthopedics, Henry M. Goldman School of Dental Medicine, Boston University, Boston, MA, United States; ^8^Center for Health Data Science, Chung Shan Medical University Hospital, Taichung, Taiwan; ^9^Institute of Medicine, Chung Shan Medical University, Taichung, Taiwan; ^10^Division of Allergy, Immunology and Rheumatology, Chung Shan Medical University Hospital, Taichung, Taiwan; ^11^Graduate Institute of Integrated Medicine, China Medical University, Taichung, Taiwan

**Keywords:** constipation, autism, microbiota, national cohort study, infant constipation, autistic spectrum disorder, National Health Insurance Research Database, gut-brain axis

## Abstract

**Background:**

Autism spectrum disorder (ASD) is a neurodevelopmental problem that presents with limited interests, repetitive behaviors, and deficits in reciprocal communication and social interactions. Mounting evidence indicates that an imbalanced gut microbiota contributes to autism *via* the gut-brain axis. Constipation may result in alteration of the gut microbiota. The clinical influence of constipation on ASD has not been fully researched. Thus, in this study we aimed to evaluate whether early childhood constipation influenced the risk of developing ASD using a nationwide population-based cohort study.

**Methods:**

We identified 12,935 constipated children aged 3 years or younger from the National Health Insurance Research Database (NHIRD) in Taiwan from 1997 to 2013. Non-constipated children were also selected from the database and propensity score matching of age, gender, and underlying comorbidities was conducted with a ratio of 1:1. Kaplan–Meier analysis was applied to determine different levels of constipation severity and cumulative incidence of autism. Subgroup analysis was also applied in this study.

**Results:**

The incidence rate of ASD was 12.36 per 100,000 person-months in the constipation group, which was higher than the rate of 7.84 per 100,000 person-months noted in the non-constipation controls. Constipated children had a significantly higher risk of autism when compared to the non-constipation group (crude relative risk = 1.458, 95% CI = 1.116–1.904; adjusted hazard ratio = 1.445, 95% CI = 1.095–1.907).

Moreover, among constipated children, a higher number of laxative prescriptions, male gender, constipation during infancy, and atopic dermatitis were significantly associated with higher risks of ASD when compared to the non-constipation group.

**Conclusion:**

Constipation in early childhood was correlated with a significantly increased risk of ASD. Clinicians should pay attention to the possibility of ASD in constipated children. Further research is necessary to study the possible pathophysiological mechanisms of this association.

## Introduction

Autism spectrum disorder (ASD) is a neurodevelopmental problem characterized by persistent deficits in reciprocal communication and social interaction. Children with ASD often present with limited interests, repetitive behavior, and varying levels of intellectual disability (1). In a recent meta-analysis, a higher prevalence was noted in boys compared with girls, with a male-to-female ratio of about 3 to 1 (2). A male-to-female ratio of 7 to 1 was found among 13,000 autistic patients in Taiwan, according to data from the Ministry of Health and Welfare in 2018 (3). Despite the increased prevalence of ASD in the past few decades, the underlying etiological factors remain unclear and there seems to be complicated interactions between genetic and environmental factors. These factors and the resultant variety of symptoms mean that the therapeutic targets are complex. Efforts are underway to find genetic or pathophysiological pathways shared by animal models and humans in order to identify a common method for better treatment targeting (4). The roles of prenatal exposure to risk factors, such as maternal-specific drug use, prenatal steroid exposure, old parental age, and use of antibiotics in the prevention of autism have been investigated in recent years (5–7). The latest studies have focused on biological molecular mechanisms, such as short chain fatty acid, which is considered to be a key connection between gut microbiota and mental diseases, brain-derived neurotrophic factor (BDNF), lipopolysaccharide (LPS), indole, as well as other immunological biomarkers appear to be involved in the biochemical mechanism of autism (8–12). However, exposure to risk factors and the mechanisms of autism in young children, especially in toddlers and infants have not been well studied yet.

Constipation is a common problem in the pediatric population. Even if constipation does not pose a serious threat to health, the related symptoms often have a highly detrimental effect on quality of life (QoL) in children, such as physical pain, emotional distress, social interaction, school life, and vitality (13). If left untreated, there may be persistent follow-up visits to the outpatient department (OPD), emergent hospital visits, and increased cost of health burden (14). In recent studies, constipation was shown to increase the risk of worsening renal function, childhood nocturnal enuresis, Parkinson’s disease, and allergic rhinitis, among other conditions (15–18). As for the treatment of constipation, prokinetic agents, osmo-or non-osmotic laxative agents, and probiotics are now used to relieve constipation and to regulate the bacterial flora in the intestinal tract (19, 20). Researchers have investigated the impact of constipation on multiple systems in our body (21). Dysbiosis may be the shared pathway between those disorders. Furthermore, the phenomenon of altered gut microbiome, if discovered to be a marked risk factor of autism, is potentially more treatable, analyzable, and preventable, compared with other disease risk factors and exposures, such as antenatal exposure, maternal metabolic condition, metabolic disorders in newborn, and genetic expression.

Dysbiosis, known as an imbalance in the composition or function of the microbial species in the intestinal tract, is believed to be one of the results of or an aggravating factor in constipation (19). It has been shown to induce alterations in the body, and to affect systemic pathways, which in turn have an impact on our central nervous system (CNS) (22, 23). The “microbiome-gut-brain axis” is known to play an important role in the pathogenesis of neurodevelopmental diseases, such as ASD (24).

Interestingly, in children with ASD, constipation is known to be a clinically significant gastrointestinal symptom, and severity of constipation has been shown to be correlated with autistic symptoms. (25–27). This correlation serves as a reminder that constipation may alter the gut microenvironment by significantly elevating the amount of specific kinds of bacteria, lowering bacterial diversity, and inducing an abnormal level of short chain fatty acids, phenomena which have been proven to be associated with ASD (28–32). Although the microbiome-gut-brain axis and its mechanisms, as well as the relationships between risk factors and diseases, including related treatments, have been extensively studied, there are no data focusing on the association between early-aged pediatric constipation and subsequent risk of an ASD diagnosis. We thus hypothesized that early childhood constipation might lead to a higher ASD risk *via* the microbiome-gut-brain axis and investigated the association between early childhood constipation and ASD by assessing a population-based retrospective, nationwide cohort from Taiwan’s National Health Institute Research Database (NHIRD).

## Materials and methods

### Data sources and study design

This was a retrospective cohort study using data collected from the National Health Insurance Research Database (NHIRD). The National Health Insurance (NHI) program provides healthcare for more than 99% of Taiwan’s population of around 23 million people. The NHIRD comprises all NHI claims data, including not only medical records of treatments and diagnoses from the outpatient department, but also documents recorded during hospitalization and in the emergency room (33). Eligible pediatric patients diagnosed with constipation during 1997–2013 were identified from the Longitudinal Health Insurance Database (LHID), which is a subset of one million subjects randomly sampled from the 23 million NHI beneficiaries in the NHIRD. Patients in the LHID are not statistically different from those in the NHIRD in terms of demographic distribution.

To guarantee the validity of these data, both autism and constipation required confirmation by not less than two OPD records or at least one inpatient discharge diagnosis, and all diagnoses were peer-reviewed. The sampled data were de-identified. The study was approved by the Institutional Review Board of Chung Shan Medical University and Hospital in Taichung, Taiwan (Approval number CS15134).

### Case definition for the constipation cohort and selection of comparisons

We retrieved insurance claim records from the LHID for the period 1997 to 2013. A diagnosis of constipation in pediatric patients was defined as an ICD-9-CM code 564.0 in two or more OPD visits or in at least one inpatient discharge note for children younger than 3 years old during the period 2001 to 2011, so as to validate the diagnosis of autism and constipation. The number of autism cases identified after early-aged constipation was compared with that in non-constipated children. We defined the first date of constipation diagnosis +1 year as the index date. For early-aged children with constipation, their history of exposure to laxatives (ATC code: A06A, A02AA02) was identified between constipation diagnosis and the index date, that was used to estimate the severity levels of constipation and the effect on the development of autism. The enrolled children were followed up until the diagnosis of autism, 31 December 2013, or withdrawal from the national health insurance system, whichever happened prior to constipation. In order to ensure that every autistic condition developed after constipation, we excluded the following children: (1) pediatric patients with missing demographics, (2) born before 2001, (3) children diagnosed with autism before the index date, (4) those whose diagnoses were made after 2011.

In order to reduce potential selection bias to a minimum, propensity score matching was utilized in this study to select non-constipation children, matching for age, gender, underlying comorbidities, and index year, in order to minimize the influences of potential confounders. The propensity score was estimated by logistic regression, and was used to match the constipation and non-constipation individuals at a ratio of 1:1. Absolute standardized difference (AbSD) was calculated to ensure that the selection of the constipation numbers and the matched non-constipation controls was not biased. When AbSD was 0.1 or less, the features of both groups could be regarded as similar. Therefore, propensity score matching was used to balance the differences of baseline features and underlying comorbidities between these two groups. Using propensity score matching, participants without current or a history of constipation were selected as controls accordingly. As antibiotics are known to have serious effects on microbiome composition, antibiotics exposure (ATC code: J01, A07A) of more than 28 days within 1 year before the index date was propensity controlled and sensitivity analyzed.

### Outcome measurements in the constipation cohort

As the primary outcome of the constipation cohort, the diagnosis of autism was retrieved using the ICD-9-CM code 299.x for ASD at two outpatient visits or in at least one inpatient discharge note. To identify the children who had higher risk of autism following constipation, subgroup analyses and multiple Cox regression of age, gender, socioeconomic status, urbanization, hospitalized stays, underlying comorbidities, and co-medication were conducted to determine any associations. Ages of constipation onset were stratified to 0–1, 1–2, and 2–3 (<3) years.

### Covariates and comorbidities adjusted by propensity score matching

The baseline features included age, sex, and underlying comorbidities, such as asthma (ICD-9-CM = 493), atopic dermatitis (ICD-9-CM = 691), preterm or low birth weight (ICD-9-CM = 765), neonatal infections (ICD-9-CM = 771), congenital malformations (ICD-9-CM = 740–759), allergic rhinitis (ICD-9-CM = 477), urticaria (ICD-9-CM = 708), intestinal infectious diseases (ICD-9-CM = 001–009), noninfective enteritis and colitis (ICD-9-CM = 555–558), and metabolic conditions [ICD-9-CM = 250, 260–269, 270–279, 774, 775 (including diabetes mellitus, nutritional deficiency, and inborn error disorders)], as well as use of co-medications that include the use of antihistamines (ATC code = R06, D04AA) and antibiotics (ATC code = J01, A07A) for ≥28 days within 1 year before the index date. The comorbidities which were noted between birth and the index date for at least one hospitalization or two outpatient visits were analyzed. After propensity score matching, the effects of the comorbidities and co-medications on the outcome were better controlled and the differences were minimized.

### Statistical analysis

Overall, using a large, national health insurance database, we retrieved data from early-aged (up to 3 years old) constipated children longitudinally, set a one-year washout period to minimize possible causes other than constipation, and compared the study group to the age-and sex-matched non-constipated children in order to determine whether early constipation increases the risk of a subsequent diagnosis of autism. The balance of baseline demographics and underlying comorbidities between the constipation group and the non-constipation group were evaluated by and presented with AbSD. In this step of analysis, the possible confounding factors were all controlled and matched. Other than basic personal and geographic data, comorbidities such as allergic diseases, infectious problems, inflammatory conditions, metabolic disorders, and certain drug exposures that were believed to have a potential impact on the mechanism of either constipation or autism were all included in the analysis. Next, the severity of constipation was stratified into three categories based on the numbers of prescriptions for laxative agents. Kaplan–Meier analysis was applied either before and after PSM to assess the cumulative incidence of autism, including the dose-defined severity analysis, and determine the causal relationship between constipation and autism. Log-rank test was utilized to calculate the significance of differences between the groups. A Cox proportional hazard model was applied to evaluate the hazard ratio of autism between the study groups. In the next step, multiple Cox regression and subgroup analysis were applied to identify the interaction within groups or between other factors and the study group. Finally, due to the marked effects that exposure to antibiotics may have on microbiome composition, which could strongly confound our primary outcome, sensitivity analysis was performed to exclude antibiotics exposure prior to the index date. The statistical software used was SAS version 9.4 (Statistical Analysis Software 9.4, SAS Institute Inc., Cary, North Carolina, United States).

## Results

### Basic demographics of the study subjects

**Figure 1:**
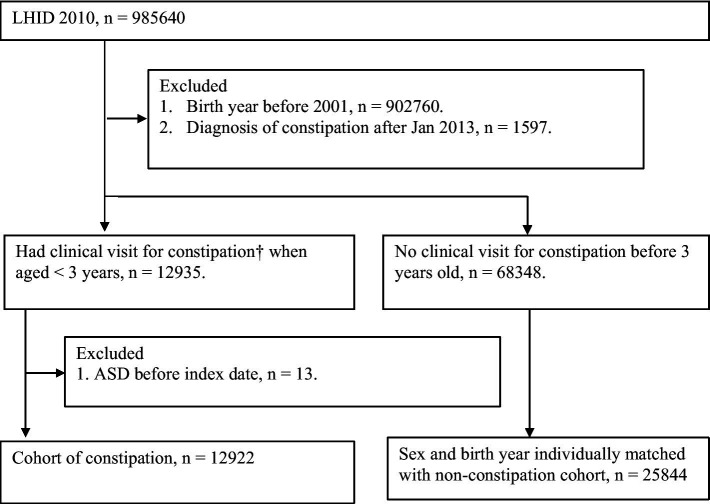
study flow chart. † Patients had constipation (ICD-9564.0, two or more OPD visits or at least one inpatient discharge note) when aged <3 years from 2001 to 2011.

A total of 12,935 children who were newly diagnosed with constipation were identified from the LHID. Patients who had been diagnosed with ASD before the index date (*n* = 13) were excluded (Figure 1). There were 12,922 constipation patients and 25,844 non-constipated patients who were matched individually by sex and birth year and selected for further analysis (Figure 1). After propensity score matching of these potential confounding factors, 12,469 subjects with constipation and an equal number of subjects without constipation were selected for inclusion in the final cohort (Table 1). Patients with constipation had a higher prevalence of asthma (14.46 vs. 10.50%, with AbSD = 0.120), atopic dermatitis (48.68 vs. 36.71%, with AbSD = 0.244), allergic rhinitis (27.60 vs. 17.88%, with AbSD = 0.131), urticaria (21.40 vs. 16.30%, with AbSD = 0.131), intestinal infectious diseases (47.11% vs. 37.63%, with AbSD = 0.193), noninfective enteritis and colitis (74.25% vs. 63.25%, with AbSD = 0.239), and metabolic conditions (28.39% vs. 19.49%, with AbSD = 0.210), as well as greater prevalence rates of antihistamines use (74.95 vs. 56.48%, with AbSD = 0.397), and antibiotics use (17.46 vs. 11.12%, with AbSD = 0.182) before propensity score matching; whereas the other comorbidities and risk factors for ASD, including preterm or low birth weight, neonatal infections, and congenital malformations of brain, were not different between the constipation and non-constipation groups (AbSD < 0.1). With respect to the personal data between the age-and sex-matched non-constipation and constipation group, geographic area distribution (AbSD = 0.130) and hospitalized stays (AbSD = 0.230) seemed to be comparable between the constipation and non-constipation group; whereas the socioeconomic status (represented by insured unit type), and urbanization did not show any differences (AbSD < 0.1). After propensity score matching, there were no statistically significant differences in any of the aforementioned possible confounding risk factors, nor were there any differences in underlying comorbidities between the constipation group and the non-constipation group (all AbSD < 0.1; Table 1).

**Table 1:** Baseline characteristics among study groups.

	Age- and sex-matched			PSM			Non-constipation	Constipation	AbSD	Non-constipation	Constipation	AbSD
N	25,844	12,922		12,469	12,469	
Sex			0.000			0.003
Female	13,738 (53.16%)	6,869 (53.16%)		6,623 (53.12%)	6,643 (53.28%)	
Male	12,106 (46.84%)	6,053 (46.84%)		5,846 (46.88%)	5,826 (46.72%)	
Age at diagnosis	0.974 ± 0.743	0.974 ± 0.743	0.000	0.983 ± 0.740	0.976 ± 0.743	0.009
0–1	7,484 (28.96%)	3,742 (28.96%)		3,524 (28.26%)	3,590 (28.79%)	
1–2	11,558 (44.72%)	5,779 (44.72%)		5,636 (45.20%)	5,587 (44.81%)	
2–3	6,802 (26.32%)	3,401 (26.32%)		3,309 (26.54%)	3,292 (26.40%)	
Insured unit type			0.054			0.058
Government	1,348 (5.22%)	614 (4.75%)		552 (4.43%)	597 (4.79%)	
Privately held company	11,872 (45.94%)	6,064 (46.93%)		5,878 (47.14%)	5,848 (46.90%)	
Agricultural organizations	1873 (7.25%)	1,093 (8.46%)		1,063 (8.53%)	1,049 (8.41%)	
Low-income	81 (0.31%)	35 (0.27%)		24 (0.19%)	35 (0.28%)	
Non-labor force	2,522 (9.76%)	1,168 (9.04%)		1,076 (8.63%)	1,128 (9.05%)	
Others	8,148 (31.53%)	3,948 (30.55%)		3,876 (31.09%)	3,812 (30.57%)	
Urbanization			0.090			0.000
Urban	15,515 (60.03%)	7,249 (56.10%)		7,085 (56.82%)	7,090 (56.86%)	
Suburban	7,999 (30.95%)	4,284 (33.15%)		4,166 (33.41%)	4,145 (33.24%)	
Rural	2,330 (9.02%)	1,389 (10.75%)		1,218 (9.77%)	1,234 (9.90%)	
Geographic area			0.130			0.069
Taipei	9,356 (36.36%)	4,080 (31.79%)		3,973 (31.86%)	4,011 (32.17%)	
North	4,282 (16.64%)	1952 (15.21%)		1917 (15.37%)	1909 (15.31%)	
Central	4,833 (18.78%)	2,745 (21.39%)		2,636 (21.14%)	2,654 (21.28%)	
South	3,313 (12.87%)	1857 (14.47%)		1810 (14.52%)	1785 (14.32%)	
Kaohsiung/Pingtung	3,423 (13.30%)	1923 (14.98%)		1898 (15.22%)	1845 (14.80%)	
East	528 (2.05%)	279 (2.17%)		235 (1.88%)	265 (2.13%)	
Baseline hospitalized stays			0.230			0.041
0 days	22,050 (85.32%)	9,772 (75.62%)		9,728 (78.02%)	9,636 (77.28%)	
1–6 days	2,751 (10.64%)	2,169 (16.79%)		1953 (15.66%)	1993 (15.98%)	
≥7 days	1,043 (4.04%)	981 (7.59%)		788 (6.32%)	840 (6.74%)	
Baseline comorbidities						
Asthma	2,713 (10.50%)	1869 (14.46%)	0.120	1,657 (13.29%)	1732 (13.89%)	0.018
Atopic dermatitis	9,488 (36.71%)	6,291 (48.68%)	0.244	6,032 (48.38%)	5,919 (47.47%)	0.018
Preterm or low birth weight	614 (2.38%)	395 (3.06%)	0.042	343 (2.75%)	372 (2.98%)	0.014
Neonatal infections	1,283 (4.96%)	924 (7.15%)	0.092	804 (6.45%)	837 (6.71%)	0.011
Congenital malformations	2,880 (11.14%)	1858 (14.38%)	0.097	1,683 (13.50%)	1722 (13.81%)	0.009
Allergic rhinitis	4,622 (17.88%)	3,566 (27.60%)	0.233	3,158 (25.33%)	3,248 (26.05%)	0.017
Urticaria	4,212 (16.30%)	2,765 (21.40%)	0.131	2,550 (20.45%)	2,580 (20.69%)	0.006
Intestinal infectious diseases	9,725 (37.63%)	6,087 (47.11%)	0.193	5,738 (46.02%)	5,737 (46.01%)	0.000
Noninfective enteritis and colitis	16,346 (63.25%)	9,594 (74.25%)	0.239	9,297 (74.56%)	9,173 (73.57%)	0.023
Metabolic conditions	5,038 (19.49%)	3,668 (28.39%)	0.210	3,222 (25.84%)	3,362 (26.96%)	0.025
Baseline co-medication						
Antihistamines	14,597 (56.48%)	9,685 (74.95%)	0.397	9,306 (74.63%)	9,252 (74.20%)	0.010
Antibiotics	2,875 (11.12%)	2,256 (17.46%)	0.182	1967 (15.78%)	2042 (16.38%)	0.016

AbSD, absolute standardized difference.

### Risk of autism in patients who had constipation

Among the 12,922 patients with constipation, 125 cases developed autism after constipation onset, which was identified in over 1,011,294 observed person-months. The incidence rate (IR) of autism was significantly higher in constipated children than in the non-constipation controls (12.36 vs. 7.84 per 100,000 person-months). Children who had constipation showed a significantly higher risk of autism compared with the non-constipation group (crude relative risk = 1.458, 95% CI = 1.116–1.904; adjusted HR = 1.431, 95% CI = 1.083–1.891, after adjusting for the confounders; Table 2). Likewise, when stratifying the severity of constipation into constipation without the need of laxatives (*n* = 4,813), with one or two laxative prescriptions (*n* = 6,553), and with over three laxative prescriptions (*n* = 1,556), it was noted that patients with more severe constipation, i.e., those receiving one or two laxative prescriptions (IR = 12.85; crude relative risk = 1.528, 95% CI = 1.099–2.123; adjusted HR = 1.517, 95% CI = 1.082–2.128) or over three laxative prescriptions (IR = 19.89; crude relative risk = 2.300, 95% CI = 1.387–3.815; adjusted HR = 2.379, 95% CI = 1.409–3.990) had a significantly higher severity-related risk of developing autism compared with the non-constipation group. The findings were similar in the PSM cohort (Table 2).

**Table 2:** Incidence of autism in study group.

	Person-months	New autism case	Incidence rate*(95% C.I.)	Crude Relative risk (95% C.I.)	Adjusted HR (95% C.I.)
Age-sex matched cohort
Non-constipation (*n* = 25,844)	2,026,818	159	7.84 (6.72–9.16)	Reference	Reference
Constipation (*n* = 12,922)	1,011,294	125	12.36 (10.37–14.73)	1.458 (1.116–1.904)	1.431 (1.083–1.891)
Constipation subgroups
Without laxatives at baseline (*n* = 4,813)	389,812	37	9.49 (6.88–13.10)	1.110 (0.733–1.682)	1.066 (0.699–1.626)
Laxatives with 1–2 prescriptions (*n* = 6,553)	505,838	65	12.85 (10.08–16.38)	1.528 (1.099–2.123)	1.517 (1.082–2.128)
Laxatives with ≥3 prescriptions (*n* = 1,556)	115,644	23	19.89 (13.22–29.93)	2.300 (1.387–3.815)	2.379 (1.419–3.990)
Propensity score-matched cohort
Non-constipation (*n* = 12,469)	977,594	48	4.91 (3.70–6.52)	Reference	Reference
Constipation (*n* = 12,469)	977,794	91	9.31 (7.58–11.43)	1.896 (1.337–2.690)	1.891 (1.333–2.684)
Constipation subgroups
Without laxatives at baseline (*n* = 4,643)	376,621	27	7.17 (4.92–10.45)	1.481 (0.924–2.373)	1.446 (0.902–2.318)
Laxatives with 1–2 prescriptions (*n* = 6,337)	490,016	48	9.80 (7.38–13.00)	1.984 (1.330–2.960)	2.002 (1.341–2.989)
Laxatives with ≥3 prescriptions (*n* = 1,489)	111,157	16	14.39 (8.82–23.50)	2.879 (1.635–5.070)	2.932 (1.660–5.180)

Adjusted HR (aHR), hazard ratio, estimated by the multiple Cox proportional hazard regression, and the covariates included sex, age, insured unit type, urbanization, geographic area, hospitalized stay, co-morbidities, and co-medication.

* Incidence rate, per 100,000 person-months.

**Figure 2:**
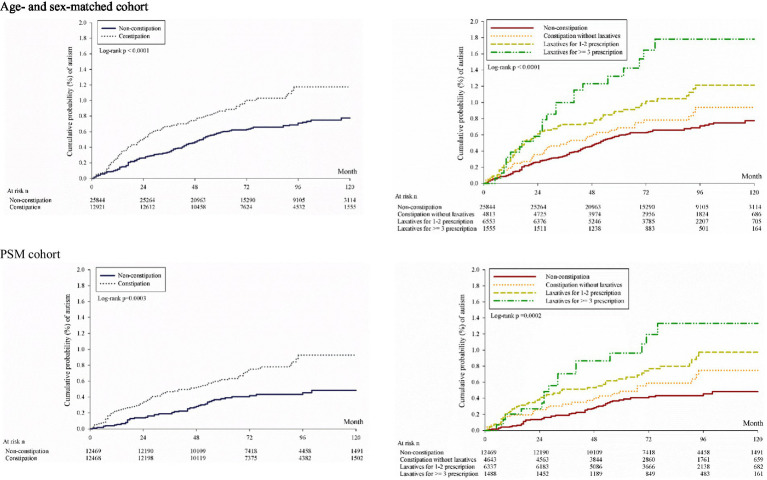
Kaplan–Meier curves of cumulative probability of development of autism. Age- and sex-matched cohort PSM cohort.

The log-rank test in the Kaplan–Meier curve analysis revealed that the cumulative risk of autism in the constipation group was significantly higher than in the non-constipation group (Figure 2); moreover, the findings were supported by the results of the log-rank test in the Kaplan–Meier curve analysis when constipation was stratified by level of severity (Figure 2). Similar findings were noted after being controlled for propensity scores. Overall, the correlation between study sets and development of autism could be observed not only in the constipation group, but also in the severity analysis of constipation (Table 2; Figure 2).

The Cox proportional hazard model revealed that regardless of the use of propensity score matching, patients with constipation who received any prescriptions for laxatives had significantly higher risk of autism compared to patients without constipation [age- and sex-matched subset (constipation with 1–2 laxatives: aHR = 1.517, 95% CI = 1.082–2.218, *p* = 0.0157; constipation with equal or more than 3 laxatives: aHR = 2.379, 95% CI = 1.419–3.990, *p* = 0.0010), PSM subset (constipation with 1–2 laxatives: aHR = 2.002, 95% CI = 1.341–2.989, *p* = 0.0007; constipation with equal or more than 3 laxatives: aHR = 2.932, 95% CI = 1.660–5.180, *p* = 0.0002)] (Table 3) [age- and sex-matched subset (constipation with 1–2 laxatives: aHR = 1.517, 95% CI = 1.082–2.218, *p* = 0.0157; constipation with equal or more than 3 laxatives: aHR = 2.379, 95% CI = 1.419–3.990, *p* = 0.0010), PSM subset (constipation with 1–2 laxatives: aHR = 2.002, 95% CI = 1.341–2.989, *p* = 0.0007; constipation with equal or more than 3 laxatives: aHR = 2.932, 95% CI = 1.660–5.180, *p* = 0.0002)] (Table 3). Male participants had a significantly higher risk of ASD compared to females (aHR = 3.700, 95% CI = 2.486–5.506, *p* < 0.0001) with or without PSM (aHR = 4.682, 95% CI = 3.351–6.541, p < 0.0001) (Table 3). Concerning the risk of ASD among all enrolled children with underlying comorbidities, congenital malformations (aHR = 1.418, 95% CI = 1.001–2.011, *p* = 0.0496), allergic rhinitis (aHR = 1.444, 95% CI = 1.043–1.999, *p* = 0.0269), and urticaria (aHR = 1.416, 95% CI = 1.029–1.949, *p* = 0.0326) were associated with higher risks of autism. After PSM, atopic dermatitis (aHR = 1.568, 95% CI = 1.109–2.218, *p* = 0.0110) and allergic rhinitis (aHR = 1.689, 95% CI = 1.160–2.458, *p* = 0.0062) were associated with higher risks of autism (Table 3). Aside from the comorbidities above, several other risk factors, namely, rural urbanization (aHR = 0.449, 95% CI = 0.221–0.912, *p* = 0.0268) and prescriptions for antihistamines (aHR = 0.648, 95% CI = 0.481–0.873, *p* = 0.0043) showed associations with a lower risk for ASD among the general population (Table 3).

**Table 3:** Multiple Cox regression for estimation of hazard ratio of autism among the age- and sex-matched and PSM cohort.

	Age-sex matched		PSM		aHR (95% C.I.)	*p*	aHR (95% C.I.)	*p*
Study group
Non-constipation	**Reference**		**Reference**	
Constipation without laxative	1.066 (0.699–1.626)	0.7675	1.446 (0.902–2.318)	0.1260
Constipation with 1–2 laxatives	1.517 (1.082–2.128)	0.0157	2.002 (1.341–2.989)	0.0007
Constipation with ≥3 laxatives	2.379 (1.419–3.990)	0.0010	2.932 (1.660–5.180)	0.0002
Sex
Female	**Reference**		**Reference**	
Male	4.682 (3.351–6.541)	<0.0001	3.700 (2.486–5.506)	<0.0001
Age at diagnosis (years)
0–1	1.310 (0.965–1.779)	0.0837	1.406 (0.956–2.070)	0.0837
1–2	**Reference**		**Reference**	
2–3	0.929 (0.649–1.330)	0.6879	0.942 (0.601–1.477)	0.7939
Insured unit type
Government	0.954 (0.538–1.691)	0.8713	0.708 (0.309–1.625)	0.4153
Privately held company	**Reference**		**Reference**	
Agricultural organizations	0.737 (0.380–1.431)	0.3672	0.566 (0.246–1.303)	0.1807
Low-income	1.443 (0.199–10.461)	0.7168	1.976 (0.267–14.613)	0.5048
Non-labor force	0.977 (0.616–1.548)	0.9201	0.609 (0.303–1.224)	0.1637
Others	1.031 (0.731–1.453)	0.8635	0.993 (0.648–1.522)	0.9755
Urbanization
Urban	**Reference**		**Reference**	
Suburban	0.840 (0.610–1.157)	0.2861	0.958 (0.647–1.419)	0.8304
Rural	0.449 (0.221–0.912)	0.0268	0.664 (0.293–1.502)	0.3250
Geographic area
Taipei	**Reference**		**Reference**	
North	0.973 (0.652–1.450)	0.8915	1.174 (0.712–1.934)	0.5304
Central	1.066 (0.716–1.588)	0.7540	1.051 (0.632–1.747)	0.8475
South	1.154 (0.742–1.794)	0.5262	1.175 (0.674–2.050)	0.5695
Kaohsiung/Pingtung	0.731 (0.452–1.182)	0.2017	0.857 (0.479–1.534)	0.6036
East	1.295 (0.512–3.276)	0.5850	1.323 (0.396–4.416)	0.6494
Baseline hospitalized stays
0 days	**Reference**		**Reference**	
1–6 days	0.847 (0.557–1.290)	0.4404	0.866 (0.535–1.400)	0.5564
≥7 days	1.084 (0.610–1.924)	0.7834	1.073 (0.554–2.076)	0.8353
Comorbidities
Asthma	0.707 (0.450–1.113)	0.1348	0.593 (0.341–1.030)	0.0636
Atopic dermatitis	1.307 (0.991–1.724)	0.0584	1.568 (1.109–2.218)	0.0110
Preterm or low birth weight	1.548 (0.822–2.913)	0.1759	1.626 (0.772–3.425)	0.2006
Neonatal infections	1.161 (0.707–1.905)	0.5558	1.205 (0.672–2.162)	0.5319
Congenital malformations	1.418 (1.001–2.011)	0.0496	1.451 (0.954–2.207)	0.0821
Allergic rhinitis	1.444 (1.043–1.999)	0.0269	1.689 (1.160–2.458)	0.0062
Urticaria	1.416 (1.029–1.949)	0.0326	1.223 (0.821–1.822)	0.3225
Intestinal infectious diseases	1.016 (0.766–1.348)	0.9110	1.047 (0.740–1.480)	0.7958
Noninfective enteritis and colitis	1.040 (0.766–1.412)	0.8022	1.120 (0.740–1.695)	0.5929
Metabolic conditions	1.181 (0.852–1.638)	0.3176	1.247 (0.847–1.836)	0.2633
Co-medication
Antihistamine	0.648 (0.481–0.873)	0.0043	0.727 (0.489–1.080)	0.1143
Antibiotics	1.033 (0.682–1.564)	0.8778	1.010 (0.623–1.636)	0.9689

### Subgroup analysis of age, gender, and time from constipation to autism onset

The subgroup analysis of the relationship between the risk of developing autism and constipation revealed a significant correlation in males (aHR = 1.386, 95% CI = 1.014–1.895), although this elevated risk was not significant in females (aHR = 1.522, 95% CI = 0.817–2.835). Likewise, the risk of ASD in the constipation group was significantly higher among patients who had constipation during infancy (aged 0–1 years; aHR = 1.704, 95% CI = 1.082–2.686). However, the risk of autism in the constipation group was not significantly higher for patients aged 1–2 years (aHR = 1.166, 95% CI = 0.745–1.823) or 2–3 years (aHR = 1.625, 95% CI = 0.896–2.946; Table 4).

**Table 4:** Subgroup analysis among the age- and sex-matched cohort.

	Non- constipation	Constipation	p for interaction
Sex			0.5072
Female (*n* = 20,607)	Reference	1.522 (0.817–2.835)	
Male (*n* = 18,159)	Reference	1.386 (1.014–1.895)	
Age at diagnosis			0.2437
0–1 (*n* = 11,226)	Reference	1.704 (1.082–2.686)	
1–2 (*n* = 17,337)	Reference	1.166 (0.745–1.823)	
2–3 (*n* = 10,203)	Reference	1.625 (0.896–2.946)	
Urbanization			0.7987
Urban (*n* = 22,764)	Reference	1.443 (1.021–2.041)	
Suburban (*n* = 12,283)	Reference	1.459 (0.882–2.414)	
Rural (*n* = 3,719)	Reference	1.098 (0.293–4.112)	
Baseline hospitalized stays			0.3004
0 day (*n* = 31,822)	Reference	1.631 (1.194–2.229)	
1–6 days (*n* = 4,920)	Reference	1.010 (0.471–2.164)	
≥7 days (*n* = 2024)	Reference	0.669 (0.235–1.906)	
Baseline co-morbidities			
Asthma			0.5811
Without (*n* = 34,184)	Reference	1.382 (1.026–1.860)	
With (*n* = 4,582)	Reference	1.816 (0.789–4.176)	
Atopic dermatitis			0.1245
Without (*n* = 22,987)	Reference	1.134 (0.753–1.707)	
With (*n* = 15,779)	Reference	1.770 (1.192–2.626)	
Preterm or low birth weight			0.8653
Without (*n* = 37,757)	Reference	1.405 (1.056–1.871)	
With (*n* = 1,009)	Reference	2.165 (0.567–8.270)	
Neonatal infections			0.9094
Without (*n* = 36,559)	Reference	1.426 (1.066–1.907)	
With (*n* = 2,207)	Reference	1.755 (0.668–4.612)	
Congenital malformations			0.4017
Without (*n* = 34,028)	Reference	1.347 (0.985–1.840)	
With (*n* = 4,738)	Reference	1.771 (0.940–3.336)	
Allergic rhinitis			0.0500
Without (*n* = 30,578)	Reference	1.170 (0.835–1.641)	
With (*n* = 8,188)	Reference	2.424 (1.412–4.161)	
Urticaria			0.3747
Without (*n* = 31,789)	Reference	1.504 (1.094–2.067)	
With (*n* = 6,977)	Reference	1.188 (0.667–2.114)	
Intestinal infectious diseases			0.2257
Without (*n* = 22,954)	Reference	1.715 (1.187–2.476)	
With (*n* = 15,812)	Reference	1.153 (0.755–1.760)	
Noninfective enteritis and colitis			0.7009
Without (*n* = 12,826)	Reference	1.393 (0.813–2.386)	
With (*n* = 25,940)	Reference	1.450 (1.046–2.011)	0.4085
Metabolic conditions			
Without (*n* = 30,060)	Reference	1.573 (1.132–2.187)	
With (*n* = 8,706)	Reference	1.110 (0.663–1.858)	
Baseline co-medication			
Antihistamine			0.3022
Without (*n* = 14,484)	Reference	1.234 (0.759–2.006)	
With (*n* = 24,282)	Reference	1.579 (1.113–2.240)	
Antibiotics			0.6598
Without (*n* = 33,635)	Reference	1.486 (1.095–2.016)	
With (*n* = 5,131)	Reference	1.066 (0.540–2.105)	

For patients with constipation, those with atopic dermatitis (aHR = 1.770, 95% CI = 1.192–2.626), allergic rhinitis (aHR = 2.424, 95% CI = 1.412–4.161), and noninfectious enteritis and colitis (aHR = 1.450, 95% CI = 1.046–2.011) were at higher risk of developing autism. Moreover, patients with constipation who used antihistamines were at high risk of developing ASD (aHR = 1.579, 95% CI = 1.113–2.240; Table 4). After testing to determine the effects of interactions among sex, age, comorbidities, antihistamines, antibiotics, and constipation on autism risk, none showed any significant interactive effect.

### Sensitivity analysis for exclusion of antibiotics exposure

As antibiotics are considered to have a notable impact on microbiome composition, subjects with antibiotics exposure prior to the index date were excluded from the analyses. The adjusted hazard ratio was estimated by multiple Cox proportional hazard regression, and the covariates included sex, age, insured unit type, urbanization, geographic area, hospitalized stay, comorbidities, and co-medication (Table 5). Even though the exclusion of some subjects reduced the number of cases, the result still showed that constipated early-aged children had significantly higher risk of autism (aHR = 1.486, 95% CI = 1.095–2.016) compared to the non-constipation group. Those receiving one or two laxative prescriptions (adjusted HR = 1.525, 95% CI = 1.048–2.220) or over three laxative prescriptions (adjusted HR = 2.355, 95% CI = 1.310–4.234) had a significantly higher severity-related risk of developing autism compared with the non-constipation group.

**Table 5:** Sensitivity analysis for exclusion of antibiotics exposure.

Exclusion of antibiotics exposure prior to index date	Adjusted HR (95% C.I.)
Non-constipation (*n* = 22,969)	Reference
Constipation (*n* = 10,666)	1.486 (1.095–2.016)
Constipation subgroups	
Without laxatives at baseline (*n* = 3,996)	1.204 (0.768–1.888)
Laxatives with 1–2 prescriptions (*n* = 5,417)	1.525 (1.048–2.220)
Laxatives with ≥3 prescriptions (*n* = 1,253)	2.355 (1.310–4.234)

Adjusted HR, hazard ratio, estimated by the multiple Cox proportional hazard regression, and the covariates included sex, age, insured unit type, urbanization, geographic area, hospitalized stay, co-morbidities, and co-medication at baseline.

## Discussion

To date and to our best knowledge, this investigation is the first, large, population-based, nationwide cohort study to evaluate the risk of autistic disorder in young pediatric patients with constipation. Higher risk of ASD was observed in constipated children, especially in more severely constipated children who received more laxative prescriptions. Other risk factors were male gender, constipation during infancy, congenital malformations, atopic dermatitis, allergic rhinitis, urticaria, and antihistamine usage. Clinicians should bear in mind that in severely constipated children, gut patency is vitally important and there is a higher risk of subsequent neurodevelopmental problems such as ASD.

It has been established over the past few decades that multiple genetic and environmental factors have a deleterious impact on gut flora, which in turn has been shown to be related to an increased risk of autism (31, 32, 34–48). Previous studies have indicated that the gut microbiome in children is still developing and is relatively unstable. The establishment of the microbiome begins from birth, with various life events causing disruptive changes in the gut microbiome, which is highly variable and develops continuously before the child reaches adulthood. Moreover, a previous study stated that there is an increased ASD risk in children with abnormal early brain structural development (39). In addition, structural and functional brain development happens mostly during the period from term birth to about 2 or 3 years old (49). This overlapping period between gut microbiota establishment and main brain volume development suggests a potential relationship between constipation and autism, which prompted the present investigation of autism in constipated children in this age group. In our study, compared with older children, constipated children younger than 1 year of age had the highest significant risk of developing ASD. We speculate that this finding may be explained, at least in part, by the immaturity of the gut microbiome and immune system in infants. It is conceivable that the earlier the development of constipation, the greater is the impact on dysbiosis and future ASD risk.

Constipation in infants and toddlers can lead to alterations of the gut microbiota, changed level of SCFA, abnormal levels of neurotransmitters, poor gut motility, and increased intestinal permeability, which through the gut-immune axis and gut-brain axis influence immune function, inflammatory processes, allergies, and metabolic conditions, potentially leading to changes in brain development with increased risk of ASD (22, 50–52). For example, Vuong and Hsiao noted that constipation can cause abnormal SCFA level, which was found to be closely related to proliferation and differentiation of immune cells and nerves, and these findings are consistent with findings reported by Stakenborg et al. and some animal models (53–55). In addition, abnormal levels of some gut microbiome-related neurotransmitters, such as serotonin and GABA, have been observed in constipated and autistic children in studies by Marler et al. and by Dinan and Cryan (56, 57). Poor motility and increased intestinal permeability were observed in constipated ASD patients (51), based on measurements of blood level of laxative agents after oral administration (58), *Lactobacillus* quantity in feces (27), and proteins of the tight junction in intestinal mucosa (40). As intestinal permeability increases, resulting in leaky gut, gut bacterial metabolites, such as lipopolysaccharide (LPS), brain-derived neurotrophic factor (BDNF), indole, may pass the mucosal barrier, causing altered cytokine levels, which then affect the brain *via* the gut-brain axis in young children (8–12, 52). Furthermore, Kang et al. achieved some success with microbiota transplant therapy in autistic children (59). In a recent study, constipation showed worsening pre-existing dysbiosis in patients with atopic diseases, such as atopic dermatitis and allergic rhinitis (14, 18). Consistent with these findings, in our study, we also found that constipated children with atopic dermatitis, allergic rhinitis, and urticaria seemed to be predisposed to subsequent ASD. Children with noninfective enteritis and colitis also had higher risk of autism. The findings suggest possible evidence of a shared or similar biophysical pathway in dysbiosis, involving a cofactor, bystander, or a crucial cause playing a role in the mechanism of autism. Hence, children with allergic diseases or an inflammatory condition should be followed up to monitor their gut microbiome composition and their developmental milestones.

It is known that exposure to antibiotics may have a serious deleterious effect on the microbiome composition, which might have markedly confounded our primary outcome, and therefore sensitivity analysis was performed to exclude subjects with antibiotics exposure lasting more than 28 days within 1 year prior to the index date. While this reduced the number of patients in the analysis, the result still showed that constipated early-aged children had significantly higher risk of future autism. Those receiving laxative prescriptions had a significantly higher severity-related risk of developing autism compared with the non-constipation group.

Our study findings imply that early childhood constipation leads to intestinal dysbiosis and the results of our analyses showed an association of constipation with future risk of ASD. During the important period when the microbiota is established, toddlers often have difficulty expressing physical discomfort. Abnormal behaviors are easily overlooked or may be incorrectly attributed to infantile colic, milk protein allergy, infantile anorexia, or failure to try soft food. Therefore, early detection of risks of dysbiosis, close monitoring of constipation, and precise evaluation of the condition of the bowels, may help to restore an imbalanced microbiome, and achieve patency and proper permeability of the guts. Education of the family and children, surveillance of developmental milestones, and referrals to a pediatric neurologist or psychiatrist should be considered when there is evidence of prodromal symptoms of ASD in young children with constipation (50).

The major advantages of this study include its large scale, with use of nationwide data analysis, detailed medical records, and a long follow-up period for both cohorts. Hence, our results can be considered reliable and selection biases were minimized. Nevertheless, there were some limitations that should be mentioned. First, the NHIRD does not contain information regarding diet exposure, schooling performance, neurological examinations, family history, personal lifestyle, genetic sequencing, or gut microbiota analysis, which may have confounded the analyses of ASD risk. Although we considered and adjusted for medications and comorbidities and used propensity scores matching to decrease the effects of possible confounding factors, these residual variables might have biased our results. Second, the diagnoses of ASD, constipation, and comorbidities were based on ICD-9 codes in this database. Thus, the validity of diagnoses could not be confirmed by reviewing personal medical documents and misclassifications may have occurred. Fortunately, these misclassifications were possibly random, and the association was often underestimated. Furthermore, an *ad hoc* committee in Taiwan has been set up to monitor claims data in Taiwan’s NHI administration to prevent any violations. Additionally, we only considered repeatedly-coded patients to improve the accuracy of these diagnoses. Finally, nearly all of the children in this study were born in Taiwan and therefore our findings might not be generalizable to other countries or ethnic groups. The authors hope that the epidemiological observations presented in this research can raise physicians’ awareness of the relationship between autism and constipation in young pediatric patients.

## Conclusion

Children with constipation in early childhood had a significantly greater risk of ASD compared with those without constipation. Clinicians should look out for prodromal symptoms of ASD in young children with constipation and be aware of the possibility of neurodevelopmental problems in these patients. Furthermore, pediatricians should assess the bowel condition, including the patency and gut microbiota, in children with ASD. Further research is needed to determine the precise pathophysiological mechanisms underlying the association between constipation and ASD.

## Data availability statement

The raw data supporting the conclusions of this article will be made available by the authors, without undue reservation.

## Author contributions

All authors listed have made a substantial, direct, and intellectual contribution to the work and approved it for publication.

## Funding

This work was supported by a grant from Taichung Veterans General Hospital Research Foundation TCVGH-1116502C and 1118701B.

## Conflict of interest

The authors declare that the research was conducted in the absence of any commercial or financial relationships that could be construed as a potential conflict of interest.

## Publisher’s note

All claims expressed in this article are solely those of the authors and do not necessarily represent those of their affiliated organizations, or those of the publisher, the editors and the reviewers. Any product that may be evaluated in this article, or claim that may be made by its manufacturer, is not guaranteed or endorsed by the publisher.
